# Different Implications of Paternal and Maternal Atopy for Perinatal IgE Production and Asthma Development

**DOI:** 10.1155/2012/132142

**Published:** 2012-01-09

**Authors:** Chih-Chiang Wu, Rong-Fu Chen, Ho-Chang Kuo

**Affiliations:** ^1^Department of Pediatrics, Show Chwan Memorial Hospital, 6 Lu-Kung Road, Lu-Kong, Changhua 50544, Taiwan; ^2^Department of Medical Research, Show Chwan Medical System in Chang Bing, Changhua 50544, Taiwan; ^3^Division of Allergy, Immunology and Rheumatology, Department of Pediatrics, Kaohsiung Chang Gung Memorial Hospital, Kaohsiung 83301, Taiwan; ^4^Chang Gung University College of Medicine, Kaohsiung 83301, Taiwan

## Abstract

Asthma is a hereditary disease associated with IgE-mediated reaction. Whether maternal atopy and paternal atopy have different impacts on perinatal IgE production and asthma development remains unclear. This paper reviews and summarizes the effects of maternal and paternal atopy on the developmental aspects of IgE production and asthma. Maternal atopy affects both pre- and postnatal IgE production, whereas paternal atopy mainly affects the latter. Maternally transmitted genes *GSTP1* and *FceRI-beta* are associated with lung function and allergic sensitization, respectively. In IgE production and asthma development, the maternal influence on gene-environment interaction is greater than paternal influence. Maternal, paternal, and/or postnatal environmental modulation of allergic responses have been linked to epigenetic mechanisms, which may be good targets for early prevention of asthma.

## 1. Introduction

The prevalence of asthma has dramatically increased over the past few decades, particularly in children [1]. Most childhood allergic diseases, such as atopic dermatitis and asthma, develop in the first few years of life [2, 3]. Understanding the developmental process of allergic diseases, which have long been attributed to IgE-mediated mechanisms [4, 5] and identifying factors that play important roles in perinatal IgE production and asthma development may help early predict and prevent the occurrence of allergic diseases. Evidence has shown that allergy sensitization may occur in fetal life [6, 7], and a number of factors have been shown to affect the development of allergic disease; family history of atopy, environmental exposure in urban areas, maternal nutritional status and stress during pregnancy, and the time and method of complementary food initiation are all potential factors that contribute to asthma. In addition, genetic polymorphisms originating in maternal or paternal inheritance have been implicated in IgE production and asthma development. This paper reviews and addresses the difference between paternal and maternal inheritance and environment in IgE production and asthma development.

## 2. Association of Antenatal IgE Production with Asthma

An increase in blood IgE levels has long been implicated in the development and severity of asthma [8, 9]. IgE production and allergy sensitization are both active processes in the prenatal and perinatal periods and are potentially influenced by genetic factors and the intrauterine and postnatal environments. Antenatal allergy sensitization with IgE production, reflected by the elevation of cord blood serum IgE (CBIgE), has been studied as a predictor of asthma and other IgE-mediated allergic diseases; however, the results are controversial (Table 1). Some studies indicate that higher CBIgE levels correlate with the development of aeroallergen sensitization [10–12], recurrent wheezing in childhood [13], later development of childhood asthma [14], and allergic rhinoconjunctivitis in adulthood [15]. However, other studies yielded discouraging results indicating that CBIgE elevation lacks the sensitivity for predicting the development of allergic diseases in childhood [16–18]. The inconsistencies may be due to differences in ethics, cut-off values of CBIgE levels, and definitions of allergic diseases in these cohorts. However, a recent study found strong evidence that maternal-fetal transfer may be a common cause of increased CBIgE levels, especially in newborns with elevated cord blood IgA levels [19] or allergen-specific IgE [20], which is not commonly found in the cord blood of newborns, suggesting maternal-fetal transfer of IgE or contamination of maternal blood. As shown in Table 1, several studies have shown a correlation between elevated CBIgE levels and allergic sensitization and/or asthma, whereas other studies revealed no correlation [10–18].

To determine whether prenatal IgE production reflects CBIgE elevation and the development of allergic diseases, we followed up a birth cohort of 230 newborns from the prenatal stage to 6 months, 18 months, 3 years, and 6 years of age. With CBIgE levels ≥0.5 kU/L considered elevated, our preliminary analysis of the total IgE levels in the 230 newborns who completed the followup revealed that newborns with elevated CBIgE (0.5 kU/L) exhibited a significantly higher risk of atopic dermatitis (odds ratio (OR), 2.067; 95% confidence interval (CI), 1.392–3.071) and allergic rhinitis (OR, 1.840; 95% CI, 1.212–2.791), but not asthma, at 6 years of age. The sensitivity and specificity of elevated CBIgE (0.5 kU/L) for predicting atopic dermatitis and allergic rhinitis were 28.5% and 83.8% and 23.9% and 85.3%, respectively. Elevated CBIgE (0.5 kU/L) was also highly correlated with elevated IgE levels at 6 years of age (150 kU/L) (OR, 2.671; 95% CI, 1.424–5.010); however, it exhibited poor sensitivity (35%) but high specificity (83.2%). This indicates that CBIgE levels are related to the development of allergic diseases and that the CBIgE prediction of allergic outcomes during childhood may be specific but not sensitive enough for clinical application. Other factors, such as elevated umbilical cord blood CCL17 levels [21], CCL22 levels [22], or reduced IFN-*γ* levels with enhanced IL-4-producing CD4+ cord blood T cells [23], have been shown to be associated with atopic dermatitis in infancy. Cord blood 25-hydroxyvitamin D levels are inversely associated with the risk of childhood wheezing [24]. More studies on the CBIgE levels and/or other chemokine or cytokine levels of cord blood are needed to improve their prediction value for childhood allergic diseases.

## 3. Different Implications of Paternal and Maternal Atopy for IgE Production and Asthma

The effect of maternal total IgE levels or atopy on cord blood IgE levels has been well recognized [25–30]; on the other hand, paternal total IgE levels and paternal atopy have little effect on antenatal IgE production or early atopy [25–29]. However, paternal total IgE level, like maternal total IgE levels, highly correlate with total IgE levels in children of preschool age [31]. In other words, maternal total IgE levels or sensitization may positively correlate with antenatal and postnatal IgE production; however, paternal total IgE levels or sensitization has little effect on antenatal IgE production but has a significant impact on the IgE production at preschool age (around 4–6 years). However, some studies have shown a poor association between parental atopy and atopic dermatitis in children up to 4 years of age [32, 33]. Although both maternal and paternal histories of asthma are associated with childhood asthma, various studies have shown that maternal history of asthma or atopy is one of the most important risk factors for childhood asthma [34–37] and is associated with increasing risk of admission for childhood asthma [38]. A 2-stage case-control study in Canada revealed that children born to asthmatic mothers are at a higher risk of developing asthma than children born to nonasthmatic mothers are (32.6% and 14.1%, resp.) [39]. Furthermore, paternal asthma is a significant and strong predictor of asthma or airway hyperresponsiveness in school-age children [40–42]. In a study on asthma in consanguineous families, paternal asthma increased the risk of asthma in both boys and girls (*P* = 0.021 for boys, *P* < 0.001 for girls), whereas maternal asthma had no significant impact on asthma in the offspring [43]. These results indicate that the effect of maternal atopy on IgE production and allergic diseases of the offspring begins at the fetal stage and continues to infancy and childhood; however, the impact of paternal atopy is not apparent until childhood.

Accumulated evidence has also shown that maternal asthma history has a greater impact on the subsequent development of allergic asthma in the offspring than paternal asthma history has. In a meta-analysis of 33 studies from 1966 to 2009 investigating the impact of maternal asthma and paternal asthma on the asthma of their offspring, the OR for asthma in children of asthmatic mothers was significantly higher than that in children of nonasthmatic mothers (3.04; 95% CI, 2.59–3.56). The corresponding OR for asthma in children of asthmatic fathers only increased to 2.44 (2.14–2.79). When comparing the OR, maternal asthma conferred a greater risk of the disease than paternal asthma did (3.04 versus 2.44, *P* = 0.037) [44]. However, some studies have shown that maternal and paternal airway hyperresponsiveness (AHR) or asthma increases the risk of AHR or asthma in their offspring [45, 46]. The opposite was observed by Kurzius-Spencer and colleagues, who showed a strong father-offspring (particularly father-son), but not mother-offspring, correlation in airway responsiveness among children [47]. Dold and colleagues have also shown that paternal asthma history has a much greater impact, with a relative risk of 4.4, than maternal asthma history has, with a 1.5-fold risk of the occurrence of wheezy bronchitis in children between the ages of 9 and 11 [48]. Similarly, in a study of 1,041 asthmatic children, Raby and his colleagues found that children with a paternal history of asthma, but not with a maternal history of asthma, showed significantly greater AHR than those without such history did [49]. Another study by Litonjua and colleagues [37] also showed that paternal contributions to the risk of childhood asthma have a greater influence on older children. These results suggest that AHR and asthma are independently inherited. Paternally derived and maternally derived asthma modulate different gene expression pathways or epigenetic mechanisms to transmit different phenotypes (AHR and asthma) in the offspring.

**Figure 1:**
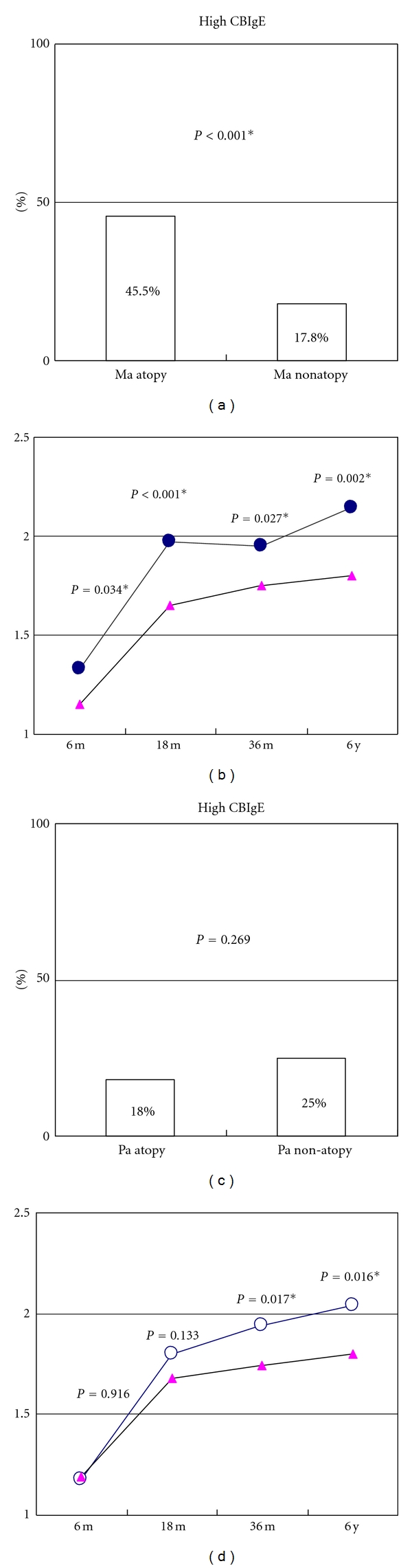
Different implications of maternal and paternal IgE levels for antenatal IgE level and postnatal IgE production at different ages in our cohort study. (a), (c): Maternal (Ma) atopy, defined as IgE > 150 kU/L, but not paternal (Pa) atopy, significantly affected antenatal IgE production, reflected by CBIgE elevation (>0.5 kU/L). (b), (d): Maternal atopy (solid circles) was significantly associated with log-transformed IgE levels at 6 months (6 m), 18 months (18 m), 3 years (36 m), and 6 years (6 y) of age, whereas paternal atopy (open circles) was only significantly associated with log-transformed levels at 3 and 6 years of age.

In our birth cohort study, we analyzed whether the presence of paternal or maternal atopy, as defined by total IgE >150 kU/L [26], influenced IgE production from the newborn stage to 6 months, 18 months, 3 years, and 6 years of age. As shown in Figure 1, maternal atopy, but not paternal atopy, significantly affected antenatal IgE production, as reflected by CBIgE elevation (Figures 1(a) and 1(c)). Maternal atopy was significantly associated with the log-transformed IgE levels at 6 months, 18 months, 3 years, and 6 years of age, whereas paternal atopy was only significantly correlated with log-transformed IgE levels at 3 years and 6 years of age (Figures 1(b) and 1(d)). These results strongly indicate that maternal influence on IgE production is exerted from the antenatal stage through childhood, whereas paternal influence on IgE production begins from early childhood and increases with increasing age.

## 4. Effects of Paternal and Maternal Inheritance and Environment on IgE Production and Asthma

Advances in the identification of asthma-susceptibility genes may provide some insights into the molecular mechanisms underlying maternal and paternal influence on childhood asthma. As shown in Table 2, Leaves and colleagues showed that a region of chromosome 7p, restricted to siblings sharing alleles inherited from fathers, not from mothers, is tightly linked with AHR in an Australian population, suggesting paternally derived alleles at this locus might affect airway responsiveness [50]. Maternal antioxidant gene polymorphisms (*GSTP1* and *GSTM1*,* GSTT1*) have been regarded as specific risk factors for asthma in the offspring and may modify the relationship between prenatal acetaminophen exposure and childhood asthma [51, 52]. Traherne and colleagues have shown that the polymorphism of the *high-affinity IgE receptor beta-chain* (*FceRI-beta*) has a strong association with positive allergy skin-prick tests and greater allergen-specific IgE levels when these polymorphisms are inherited from the mothers [53]. Cookson and his colleagues found that the transmission of atopy at the chromosome 11q13 allele is detectable only through the maternal line [54]. These parent-derived alleles associated with IgE production and asthma provide insights into the impact of gender on the inheritance of IgE production and the development of asthma.

There are several possible reasons to explain why mothers and fathers have different impacts on IgE production and asthma development in their offspring, including exclusive exposure to maternal environmental factors during fetal development, fetomaternal-shared perinatal environmental exposures (including breastfeeding), different hormones, and distinct genetic imprinting [49]. Moreover, certain genetic alleles may have sex-specific effects and may be expressed to a greater level in male or female individuals, thereby making more specific contributions when inherited from the mother or father.

Besides inheritance, maternal and paternal environment can affect the development of asthma differently. Several lines of evidence have indicated that factors present during fetal development influence immune responses and allergen sensitization in early life. During pregnancy, the fetomaternal interface is surrounded by Th2-prone environment [55], which may suppress fetus-directed maternal Th1 immune responses [56]. This Th2 environment may be a good niche for fetal allergy sensitization, because the Th2 cytokine microenvironment may prime T cells toward allergic differentiation [57]. Maternal environmental factors, including maternal prenatal exposure to tobacco smoke [58–62], household allergens [63], traffic air pollution [64], and maternal occupational exposure to latex and biocides [65], are associated with childhood asthma; however, increasing maternal age, maternal prenatal exposure to cat or dog [58, 66], maternal fish intake [67], and maternal exposure to farming environments [68] have been shown to exert protective effects. One study revealed that folic acid supplements during pregnancy are associated with a slightly increased risk of wheezing and lower respiratory tract infections up to 18 months of age and suggests that methyl-group donors in the maternal diet during pregnancy influence respiratory health in children via epigenetic mechanisms [69]. Another study showed that serum folate levels are inversely associated with total IgE levels and that a dose-response relationship exists between serum folate and outcomes of high total IgE level, atopy, and wheezing [70]. However, a recent study does not support the relationship between folic acid supplement and asthma development [71]. In contrast, fewer paternal environmental factors are associated with IgE production and asthma development. It has been shown that paternal occupational flour dust exposure was associated with the development of asthma [65]. These results indicate that environmental factors during pregnancy, directly or indirectly, play important roles in allergic sensitization or disease development.

Environmental factors may influence gene expression, cytokine secretion, T-cell differentiation, and the development of allergic diseases via epigenetic mechanisms, possibly by DNA methylation and histone modification. In a study using the *Aspergillus fumigatus* allergen murine model, chronic inhalation of diesel exhaust particles induced hypermethylation at the CpG-45, CpG-53, and CpG-205 sites of the *IFN-γ* promoter in CD4 cells, and hypomethylation at CpG-408 in the proximal* IL-4 *promoter in CD4 cells, both of which significantly correlated with higher IgE production [74]. Perera and colleagues found that transplacental exposure to traffic-related polycyclic aromatic hydrocarbons (PAHs) is significantly associated with hypermethylation of *acyl-CoA synthetase long-chain family member 3* (*ACSL3*) [75], which may diminish fatty acid utilization and possibly influence membrane phospholipid composition. Whether these functional changes directly affect the development of the asthmatic phenotype is unknown and deserves further investigation.

## 5. Paternal and Maternal Influence on Gene-Environment Interactions in Perinatal IgE Production and Childhood Asthma

IgE is produced by activated B cells, which interact with Th2 cells and undergo isotype class switching after the induction of Th2 cell-derived cytokines, particularly *IL-4* and *IL-13*. Some lines of evidence indicate that IgE production in children and adults is under strong genetic control [76, 77], with heritability ranging from 60% to 87% in childhood. We previously found that 21 SNPs in 14 allergy candidate genes on chromosomes 4, 5, 6, 9, 10, 11, 12, 16, and 20 are associated with elevated levels of CBIgE [78], a finding similar to findings of the studies on genetic association of serum IgE and asthma [79, 80].

We have also previously shown the effects of gene-gene (IL13, rs20541 interaction between *CCL17*, rs223900, and *CXCL10*, rs867562 on antenatal IgE production) and gene-environment (maternal atopy alone and its interaction with *PIM1, GPIAP1, NOS2A, CTLA4, ADAM33, LTA, PDE2A, GSR, IL13, FGF, CCL22, *or *CAT* can affect antenatal IgE production) interactions on CBIgE production [78]. Another study in Australia made a similar observation that genetic variants in the Th2 pathways, particularly in the *IL-13, IL-13RA1, *and *STAT6* genes, are significantly associated with CBIgE concentration individually and jointly. The gene-gene interaction and ethnic heterogeneity observed in that study are similar to those observed in our study [93]. Both studies indicate that genetic regulation of IgE production begins in the prenatal stage and is influenced by different genetic backgrounds and maternal atopy status via gene-environment interactions.

In IgE production and asthma, the maternal influence on gene-environment interaction is more prominent than the paternal influence is. Several studies have supported the gender-dependent gene-environment interactions on asthma development, such as polymorphisms of *CTLA-4* [26], *GSTM1* [94, 95], *GSTP1* [96], *IL-13* [97], *IL-1Ra* [98], *βAR* [99], *TGF-beta1* [100], *HLA-G* [101, 102], *CD14* [103–106], and *TLR2* [107], may influence the development of asthma through interactions with other maternal environmental factors, such as maternal tobacco smoke exposure, maternal atopy, or maternal prenatal exposure to a farming environment. Sensitization of the fetus due to interactions between exclusive in utero maternal environmental factors and fetus susceptible genes may be the most important reason why maternal influence is greater than paternal influence during fetal and infancy periods.

 Multiple postnatal environmental factors have been proven to be associated with increased risk of childhood asthma. As shown in Table 3, passive exposure to tobacco smoke increases the risk of allergen sensitization [81] and childhood rhinitis and asthma [60, 82–84], particularly when mothers are not atopic. Children exposed to traffic exhaust have increased risk of recurrent night cough and wheezing [85] and allergen sensitization in children with specific genetic polymorphisms [64], which may be associated with IgE-mediated asthma. In urban areas with high levels of vehicular traffic, the most abundant components of air pollution are airborne particulate matter, nitrogen dioxide, and ozone [86], which may explain the increased prevalence of asthma or allergic respiratory tract diseases in urban areas. Recent evidence has also revealed that allergic sensitization is positively linked to rhinovirus-related wheezing but not wheezing caused by other viruses [89]. Early exposure to acetaminophen [90] and broad-spectrum antibiotics [91] are also weakly associated with childhood wheezing or asthma. On the other hand, breastfeeding [87, 88] and exposure to farming environment and animals with increased levels of microbial substances may protect against IgE-mediated allergic diseases [68]. A report of two prospective birth cohorts on the association of complementary foods with allergy stated that introduction of solid foods with a high diversity of different solids before the end of the fourth month may increase the risk of later allergy, particularly eczema. However, delayed introduction of solid foods beyond the sixth month of age or the avoidance of allergenic foods during the first year does not prevent allergy development [67]. These findings are also reflected in the new recommendation for allergy prevention published by the American Academy of Pediatrics [108] and the European Society of Pediatric Gastroenterology, Hepatology and Nutrition [109]. Joseph and his colleagues reported a different finding that early introduction of complementary food before the age of 4 months is associated with a reduced risk of peanut (and perhaps egg) sensitization by the age of 2 to 3 years but only for children with a parental history of asthma or allergy [92]. Whether paternal or maternal atopy affects the postnatal environmental modulation of IgE production and asthma development remains to be determined.

## 6. Conclusions

The development of immunity and allergen sensitization is believed to start during the fetal period. CBIgE levels, reflecting antenatal IgE production, is significantly associated with total IgE levels, allergen-specific IgE levels, and even occurrence of asthma, according to most studies. However, the sensitivity and negative prediction rate are still unsatisfactory, making it a poor predictor of childhood asthma. Postnatal IgE, particularly after early childhood, is more sensitive and more relevant to clinical applications in aiding the diagnosis of IgE-medicated allergic diseases. The improvement of CBIgE application methods for predicting allergic diseases remains a big challenge.

Most studies have shown that both paternal and maternal factors have great impacts on IgE production and asthma development in the offspring. Genetic and environmental factors from both parents also contribute to this impact. This literature review revealed that maternal influence begins in the fetus and continues through infancy, childhood, and even adulthood, whereas paternal effect may not be apparent until early childhood, and the effect may increase with increasing age.

Asthma is a complex disease involving multiple genetic backgrounds and multiple environmental insults. Some specific genetic alleles may display greater effect in specific environments. Interactions of maternal oxidative stress genes (*GSTM1, GSTP1, CAT*, and *MPO*) with maternal prenatal exposure to air pollutants or tobacco smoke may contribute to asthma or allergic airway responsiveness. On the other hand, interactions of *TLR2, CD14* genotype, with farm exposure, and/or endotoxin exposure may protect against the the development of asthma. Experimental studies have revealed that most environmental factors manifest their effects on asthma development through epigenetic mechanisms, such as DNA methylation and histone modification. An increase in CpG methylation of the *IFN-γ* promoter and decrease in CpG methylation of the *IL-4* promoter have been observed in response to chronic exposure to diesel exhaust [74], and maternal prenatal exposure to PAHs has been linked to hypermethylation of *ACSL3* [75]. More studies are warranted to investigate the gene-environment interactions among maternal inheritance, paternal inheritance, and environment in perinatal stages for the prevention of IgE production and asthma development.

## Figures and Tables

**Table 1:** Does CBIgE elevation predict allergy?

Parameters	Population studied, country	Reference
Parameters correlated with elevated CBIgE		

Skin-prick test at age 4 yr	1456, England	[10]
Allergic sensitization and recurrent wheezing at age 7 yr	380 high-risk newborns, Canada	[13]
Allergic sensitization at 4 and 10 yr and asthma at 10 yr	1456, USA	[14]
Skin-prick test at age 5 yr, allergic rhinoconjunctivitis at age 20 yr, and total IgE at ages 11 and 20 yr	200, Finland	[15]
High IgE and allergic sensitization between the ages of 18 and 24 months	1884, Sweden	[11]
Total IgE and allergic diseases before 5 years of age	1884, USA	[12]

Parameters not correlated with elevated CBIgE		

No significant association with recurrent wheezing	1314, Germany	[16]
Not better than family history to predict infant atopy	2814, USA	[17]
Family history of atopy far more sensitive than CBIgE	1111, UK	[18]

**Table 2:** Effect of parental background on IgE production and asthma development.

	Maternal background	Paternal background
Gene	Maternal antioxidant gene polymorphisms: GSTP1 [51] GSTM1 and GSTT1 [52] Polymorphism of the beta-chain of high-affinity IgE receptor (FceRI-beta) [53], and 11q13 allele [54]	An allele at chromosome 7p [50]

Environment	Maternal asthma [34–39] Fetal exposure to tobacco smoke, household allergens, and latex and/or biocides [58–63, 65] Fetal exposure to traffic air pollution [64] Maternal prenatal exposure to farm, farm animals, and cat or dog [58, 66, 68] Mediterranean diet, fish intake, fatty acid status, and folic acid supplements during pregnancy [24, 69, 72, 73]	Paternal asthma [40–43] Paternal occupational flour dust exposure [65]

**Table 3:** Postnatal environmental factors associated with risk of childhood asthma.

Increased risk of childhood asthma	Decreased risk of childhood asthma
Environmental tobacco smoke exposure [60, 81–84]	Exposure to a farming environment [68]
Exposure to traffic exhaust and air pollution [64, 85, 86]	Breastfeeding [87, 88]
Rhinovirus-related wheezing [89]	
Early exposure to acetaminophen [90]	
Broad spectrum antibiotics used in early childhood [91]	
Early introduction of solid diet at infancy [67, 92]	

## References

[1] Pedersen SE, Hurd SS, Lemanske RF (2011). Global strategy for the diagnosis and management of asthma in children 5 years and younger. *Pediatric Pulmonology*.

[2] Kulig M, Bergmann R, Klettke U, Wahn V, Tacke U, Wahn U (1999). Natural course of sensitization to food and inhalant allergens during the first 6 years of life. *The Journal of Allergy and Clinical Immunology*.

[3] Spergel JM, Paller AS (2003). Atopic dermatitis and the atopic march. *The Journal of Allergy and Clinical Immunology*.

[4] Vervloet D, Bongrand P, Charpin J (1978). Absolute determination of IgE antibodies to grass pollen allergens. *Allergy*.

[5] Burrows B, Marinez FD, Halonen M, Barbee RA, Cline MG (1989). Association of asthma with serum IgE levels and skin-test reactivity to allergens. *The New England Journal of Medicine*.

[6] Kihlström A, Lilja G, Pershagen G, Hedlin G (2003). Exposure to high doses of birch pollen during pregnancy, and risk of sensitization and atopic disease in the child. *Allergy*.

[7] Boyle RJ, Tang MLK (2006). Can allergic diseases be prevented prenatally?. *Allergy*.

[8] Sandeep T, Roopakala MS, Silvia CRWD, Chandrashekara S, Rao M (2010). Evaluation of serum immunoglobulin e levels in bronchial asthma. *Lung India*.

[9] Satwani H, Rehman A, Ashraf S, Hassan A (2009). Is serum total IgE levels a good predictor of allergies in children?. *The Journal of the Pakistan Medical Association*.

[10] Karmaus W, Arshad H, Mattes J (2001). Does the sibling effect have its origin in utero? Investigating birth order, cord blood immunoglobulin E concentration, and allergic sensitization at age 4 years. *American Journal of Epidemiology*.

[11] Croner S, Kjellman NIM, Eriksson B, Roth A (1982). IgE screening in 1701 newborn infants and the development of atopic disease during infancy. *Archives of Disease in Childhood*.

[12] Hansen LG, Halken S, Host A, Moller K, Osterballe O (1993). Prediction of allergy from family history and cord blood IgE levels. A follow-up at the age of 5 years. Cord blood IgE. IV. *Pediatric Allergy and Immunology*.

[13] Ferguson A, Dimich-Ward H, Becker A (2009). Elevated cord blood IgE is associated with recurrent wheeze and atopy at 7 yrs in a high risk cohort. *Pediatric Allergy and Immunology*.

[14] Sadeghnejad A, Karmaus W, Davis S, Kurukulaaratchy RJ, Matthews S, Arshad SH (2004). Raised cord serum immunoglobulin E increases the risk of allergic sensitisation at ages 4 and 10 and asthma at age 10. *Thorax*.

[15] Pesonen M, Kallio MJT, Siimes MA, Elg P, Björksten F, Ranki A (2009). Cord serum immunoglobulin E as a risk factor for allergic symptoms and sensitization in children and young adults. *Pediatric Allergy and Immunology*.

[16] Edenharter G, Bergmann RL, Bergmann KE (1998). Cord blood-IgE as risk factor and predictor for atopic diseases. *Clinical and Experimental Allergy*.

[17] Hansen LG, Host A, Halken S (1992). Cord blood IgE. II. Prediction of atopic disease. A follow-up at the age of 18 months. *Allergy*.

[18] Hide DW, Arshad SH, Twiselton R, Stevens M (1991). Cord serum IgE: an insensitive method for prediction of atopy. *Clinical and Experimental Allergy*.

[19] Ownby DR, McCullough J, Johnson CC, Peterson EL (1996). Evaluation of IgA measurements as a method for detecting maternal blood contamination of cord blood samples. *Pediatric Allergy and Immunology*.

[20] Bønnelykke K, Pipper CB, Bisgaard H (2010). Transfer of maternal IgE can be a common cause of increased IgE levels in cord blood. *The Journal of Allergy and Clinical Immunology*.

[21] Miyahara H, Okazaki N, Nagakura T, Korematsu S, Izumi T (2011). Elevated umbilical cord serum TARC/CCL17 levels predict the development of atopic dermatitis in infancy. *Clinical and Experimental Allergy*.

[22] Sandberg M, Frykman A, Ernerudh J (2009). Cord blood cytokines and chemokines and development of allergic disease. *Pediatric Allergy and Immunology*.

[23] Herberth G, Heinrich J, Röder S (2010). Reduced IFN-*γ*- and enhanced IL-4-producing CD4+ cord blood T cells are associated with a higher risk for atopic dermatitis during the first 2 yr of life. *Pediatric Allergy and Immunology*.

[24] Camargo CA, Ingham T, Wickens K (2011). Cord-blood 25-hydroxyvitamin D levels and risk of respiratory infection, wheezing, and asthma. *Pediatrics*.

[25] Liu CA, Wang CL, Chuang H, Ou CY, Hsu TY, Yang KD (2003). Prenatal prediction of infant atopy by maternal but not paternal total IgE levels. *The Journal of Allergy and Clinical Immunology*.

[26] Yang KD, Ou CY, Hsu TY (2007). Interaction of maternal atopy, CTLA-4 gene polymorphism and gender on antenatal immunoglobulin E production. *Clinical and Experimental Allergy*.

[27] Shah S, Bapat MM (2006). Parental history of allergy, maternal serum IigE & cord serum IgE. *Indian Journal of Medical Sciences*.

[28] Johnson CC, Ownby DR, Peterson EL (1996). Parental history of atopic disease and concentration of cord blood IgE. *Clinical and Experimental Allergy*.

[29] Magnusson CGM (1988). Cord serum IgE in relation to family history and as predictor of atopic disease in early infancy. *Allergy*.

[30] Oryszczyn MP, Annesi-Maesano I, Campagna D, Sahuquillo J, Huel G, Kauffmann F (1999). Head circumference at birth and maternal factors related to cord blood total IgE. *Clinical and Experimental Allergy*.

[31] Canfield SM, Jacobson JS, Perzanowski MS (2008). Total and specific IgE associations between New York City Head Start children and their parents. *The Journal of Allergy and Clinical Immunology*.

[32] Böhme M, Wickman M, Nordvall SL, Svartengren M, Wahlgren CF (2003). Family history and risk of atopic dermatitis in children up to 4 years. *Clinical and Experimental Allergy*.

[33] Chang WT, Sun HL, Lue KH, Chou MC (2005). Predictability of early onset atopic dermatitis by cord blood IgE and parental history. *Acta Paediatrica Taiwanica*.

[34] Bracken MB, Belanger K, Cookson WO, Triche E, Christiani DC, Leaderer BP (2002). Genetic and perinatal risk factors for asthma onset and severity: a review and theoretical analysis. *Epidemiologic Reviews*.

[35] Johnson CC, Ownby DR, Zoratti EM, Alford SH, Williams LK, Joseph CLM (2002). Environmental epidemiology of pediatric asthma and allergy. *Epidemiologic Reviews*.

[36] Aberg N (1993). Familial occurrence of atopic disease: genetic versus environmental factors. *Clinical and Experimental Allergy*.

[37] Litonjua AA, Carey VJ, Burge HA, Weiss ST, Gold DR (1998). Parental history and the risk for childhood asthma: does mother confer more risk than father?. *American Journal of Respiratory and Critical Care Medicine*.

[38] Davidson R, Roberts SE, Wotton CJ, Goldacre MJ (2010). Influence of maternal and perinatal factors on subsequent hospitalisation for asthma in children: evidence from the Oxford record linkage study. *BMC Pulmonary Medicine*.

[39] Martel MJ, Rey E, Malo JL (2009). Determinants of the incidence of childhood asthma: a two-stage case-control study. *American Journal of Epidemiology*.

[40] Klinnert MD, Nelson HS, Price MR, Adinoff AD, Leung DY, Mrazek DA (2001). Onset and persistence of childhood asthma: predictors from infancy. *Pediatrics*.

[41] Morais-Almeida M, Caspar A, Pires G, Prates S, Rosado-Pinto J (2007). Risk factors for asthma symptoms at school age: an 8-year prospective study. *Allergy & Asthma Proceedings*.

[42] Ly NP, Soto-Quirós ME, Avila L (2008). Paternal asthma, mold exposure, and increased airway responsiveness among children with asthma in Costa Rica. *Chest*.

[43] Joseph M, Zoubeidi T, Al-Dhaheri SM (2009). Paternal asthma is a predictor for childhood asthma in the consanguineous families from the United Arab Emirates. *Journal of Asthma*.

[44] Lim RH, Kobzik L, Dahl M (2010). Risk for asthma in offspring of asthmatic mothers versus fathers: a meta-analysis. *PLoS One*.

[45] Gray L, Peat JK, Belousova E, Xuan W, Woolcock AJ (2000). Family patterns of asthma, atopy and airway hyperresponsiveness: an epidemiological study. *Clinical and Experimental Allergy*.

[46] Jenkins MA, Hopper JL, Bowes G, Carlin JB, Flander LB, Giles GG (1994). Factors in childhood as predictors of asthma in adult life. *British Medical Journal*.

[47] Kurzius-Spencer M, Holberg CJ, Sherrill DL (2004). Segregation analysis of bronchial hyperresponsiveness in a general population in north Italy. *American Journal of Medical Genetics A*.

[48] Dold S, Wjst M, von Mutius E, Reitmeir P, Stiepel E (1992). Genetic risk for asthma, allergic rhinits, and atopic dermatitis. *Archives of Disease in Childhood*.

[49] Raby BA, Van Steen K, Celedón JC, Litonjua AA, Lange C, Weiss ST (2005). Paternal history of asthma and airway responsiveness in children with asthma. *American Journal of Respiratory and Critical Care Medicine*.

[50] Leaves NI, Bhattacharyya S, Wiltshire S, Cookson WOCM (2002). A detailed genetic map of the chromosome 7 bronchial hyper-responsiveness locus. *European Journal of Human Genetics*.

[51] Carroll WD, Lenney W, Child F, Strange RC, Jones PW, Fryer AA (2005). Maternal glutathione S-transferase GSTP1 genotype is a specific predictor of phenotype in children with asthma. *Pediatric Allergy and Immunology*.

[52] Shaheen SO, Newson RB, Ring SM, Rose-Zerilli MJ, Holloway JW, Henderson AJ (2010). Prenatal and infant acetaminophen exposure, antioxidant gene polymorphisms, and childhood asthma. *The Journal of Allergy and Clinical Immunology*.

[53] Traherne JA, Hill MR, Hysi P (2003). LD mapping of maternally and non-maternally derived alleles and atopy in FcERI-*β*. *Human Molecular Genetics*.

[54] Cookson WOCM, Young RP, Sandford AJ (1992). Maternal inheritance of atopic IgE responsiveness on chromosome 11q. *The Lancet*.

[55] Wegmann TG, Lin H, Guilbert L, Mosmann TR (1993). Bidirectional cytokine interactions in the maternal-fetal relationship: is successful pregnancy a TH2 phenomenon?. *Immunology Today*.

[56] Piccinni MP, Beloni L, Livi C, Maggi E, Scarselli G, Romagnani S (1998). Defective production of both leukemia inhibitory factor and type 2 T- helper cytokines by decidual T cells in unexplained recurrent abortions. *Nature Medicine*.

[57] Demeure CE, Wu CY, Shu U (1994). *In vitro* maturation of human neonatal CD4 T lymphocytes: II. Cytokines present at priming modulate the development of lymphokine production. *Journal of Immunology*.

[58] Sybilski AJ, Doboszynska A, Samolinski B (2009). Total and antigen-specific IGE levels in umbilical cord blood. *European Journal of Medical Research*.

[59] Magnusson LL, Olesen AB, Wennborg H, Olsen J (2005). Wheezing, asthma, hayfever, and atopic eczema in childhood following exposure to tobacco smoke in fetal life. *Clinical and Experimental Allergy*.

[60] Radic SD, Gvozdenovic BS, Pesic IM, Zivkovic ZM, Skodric-Trifunovic V (2011). Exposure to tobacco smoke among asthmatic children: parents' smoking habits and level of education. *The International Journal of Tuberculosis and Lung Disease*.

[61] Keil T, Lau S, Roll S (2009). Maternal smoking increases risk of allergic sensitization and wheezing only in children with allergic predisposition: longitudinal analysis from birth to 10 years. *Allergy*.

[62] Zlotkowska R, Zejda JE (2005). Fetal and postnatal exposure to tobacco smoke and respiratory health in children. *European Journal of Epidemiology*.

[63] Peters JL, Suglia SF, Platts-Mills TAE, Hosen J, Gold DR, Wright RJ (2009). Relationships among prenatal aeroallergen exposure and maternal and cord blood IgE: project ACCESS. *The Journal of Allergy and Clinical Immunology*.

[64] Braback L, Forsberg B (2009). Does traffic exhaust contribute to the development of asthma and allergic sensitization in children: findings from recent cohort studies. *Environmental Health*.

[65] Tagiyeva N, Devereux G, Semple S (2010). Parental occupation is a risk factor for childhood wheeze and asthma. *The European Respiratory Journal*.

[66] Aichbhaumik N, Zoratti EM, Strickler R (2008). Prenatal exposure to household pets influences fetal immunoglobulin e production. *Clinical and Experimental Allergy*.

[67] Sausenthaler S, Heinrich J, Koletzko S (2011). Early diet and the risk of allergy: what can we learn from the prospective birth cohort studies GINIplus and LISAplus?. *The American Journal of Clinical Nutrition*.

[68] von Mutius E, Radon K (2008). Living on a farm: impact on asthma induction and clinical course. *Immunology and Allergy Clinics of North America*.

[69] Håberg SE, London SJ, Stigum H, Nafstad P, Nystad W (2009). Folic acid supplements in pregnancy and early childhood respiratory health. *Archives of Disease in Childhood*.

[70] Matsui EC, Matsui W (2009). Higher serum folate levels are associated with a lower risk of atopy and wheeze. *The Journal of Allergy and Clinical Immunology*.

[71] Magdelijns FJH, Mommers M, Penders J, Smits L, Thijs C (2011). Folic acid use in pregnancy and the development of atopy, asthma, and lung function in childhood. *Pediatrics*.

[72] Notenboom ML, Mommers M, Jansen EHJM, Penders J, Thijs C (2011). Maternal fatty acid status in pregnancy and childhood atopic manifestations: KOALA Birth Cohort Study. *Clinical and Experimental Allergy*.

[73] Bloomberg GR (2011). The influence of environment, as represented by diet and air pollution, upon incidence and prevalence of wheezing illnesses in young children. *Current Opinion in Allergy and Clinical Immunology*.

[74] Liu J, Ballaney M, Al-Alem U (2008). Combined inhaled diesel exhaust particles and allergen exposure alter methylation of T helper genes and Ige production *in vivo*. *Toxicological Sciences*.

[75] Perera F, Tang W-Y, Herbstman J (2009). Relation of DNA methylation of 5′-CpG island of ACSL3 to transplacental exposure to airborne polycyclic aromatic hydrocarbons and childhood asthma. *PLoS One*.

[76] Jacobsen HP, Herskind AM, Nielsen BW, Husby S (2001). IgE in unselected like-sexed monozygotic and dizygotic twins at birth and at 6 to 9 years of age: high but dissimilar genetic influence on IgE levels. *The Journal of Allergy and Clinical Immunology*.

[77] Palmer LJ, Burton PR, Faux JA, James AL, Musk AW, Cookson WOCM (2000). Independent inheritance of serum Immunoglobulin E concentrations and airway responsiveness. *American Journal of Respiratory and Critical Care Medicine*.

[78] Yang KD, Chang JC, Chuang H (2010). Gene-gene and gene-environment interactions on IgE production in prenatal stage. *Allergy*.

[79] Chang JC, Liu CA, Chuang H (2004). Gender-limited association of cytotoxic T-lymphocyte antigen-4 (CTLA-4) polymorphism with cord blood IgE levels. *Pediatric Allergy and Immunology*.

[80] Malerba G, Pignatti PF (2005). A review of asthma genetics: gene expression studies and recent candidates. *Journal of Applied Genetics*.

[81] Raherison C, Pénard-Morand C, Moreau D (2008). Smoking exposure and allergic sensitization in children according to maternal allergies. *Annals of Allergy, Asthma & Immunology*.

[82] Zuraimi MS, Tham KW, Chew FT, Ooi PL, David K (2008). Home exposures to environmental tobacco smoke and allergic symptoms among young children in Singapore. *International Archives of Allergy and Immunology*.

[83] Tanaka K, Miyake Y, Arakawa M, Sasaki S, Ohya Y (2007). Prevalence of asthma and wheeze in relation to passive smoking in Japanese children. *Annals of Epidemiology*.

[84] Tanaka K, Miyake Y, Sasaki S, Ohya Y, Hirota Y (2008). Maternal smoking and environmental tobacco smoke exposure and the risk of allergic diseases in Japanese infants: the Osaka maternal and child health study. *Journal of Asthma*.

[85] Sucharew H, Ryan PH, Bernstein D (2010). Exposure to traffic exhaust and night cough during early childhood: the CCAAPS birth cohort. *Pediatric Allergy and Immunology*.

[86] D’Amato G, Cecchi L, D’Amato M, Liccardi G (2010). Urban air pollution and climate change as environmental risk factors of respiratory allergy: an update. *Journal of Investigational Allergology and Clinical Immunology*.

[87] Tanaka K, Miyake Y, Sasaki S (2010). Association between breastfeeding and allergic disorders in Japanese children. *International Journal of Tuberculosis and Lung Disease*.

[88] Scholtens S, Wijga AH, Brunekreef B (2009). Breast feeding, parental allergy and asthma in children followed for 8 years. The PIAMA birth cohort study. *Thorax*.

[89] Jartti T, Kuusipalo H, Vuorinen T (2010). Allergic sensitization is associated with rhinovirus-, but not other virus-, induced wheezing in children. *Pediatric Allergy and Immunology*.

[90] Garcia-Marcos L, Sanchez-Solis M, Perez-Fernandez V (2011). Early exposure to acetaminophen and allergic disorders. *Current Opinion in Allergy and Clinical Immunology*.

[91] Jedrychowski W, Perera F, Maugeri U (2011). Wheezing and asthmamay be enhanced by broad spectrum antibiotics used in early childhood. concept and results of a pharmacoepidemiology study. *Journal of Physiology and Pharmacology*.

[92] Joseph CLM, Ownby DR, Havstad SL (2011). Early complementary feeding and risk of food sensitization in a birth cohort. *The Journal of Allergy and Clinical Immunology*.

[93] Hong X, Tsai HJ, Liu X (2010). Does genetic regulation of IgE begin in utero? Evidence from T H1/TH2 gene polymorphisms and cord blood total IgE. *The Journal of Allergy and Clinical Immunology*.

[94] Gilliland FD, Li YF, Dubeau L (2002). Effects of glutathione S-transferase M1, maternal smoking during pregnancy, and environmental tobacco smoke on asthma and wheezing in children. *American Journal of Respiratory and Critical Care Medicine*.

[95] Rogers AJ, Brasch-Andersen C, Ionita-Laza I (2009). The interaction of glutathione S-transferase M1-null variants with tobacco smoke exposure and the development of childhood asthma. *Clinical and Experimental Allergy*.

[96] Perzanowski MS, Miller RL, Tang D (2010). Prenatal acetaminophen exposure and risk of wheeze at age 5 years in an urban low-income cohort. *Thorax*.

[97] Sadeghnejad A, Karmaus W, Arshad SH, Kurukulaaratchy R, Huebner M, Ewart S (2008). IL13 gene polymorphisms modify the effect of exposure to tobacco smoke on persistent wheeze and asthma in childhood, a longitudinal study. *Respiratory Research*.

[98] Ramadas RA, Sadeghnejad A, Karmaus W (2007). Interleukin-1R antagonist gene and pre-natal smoke exposure are associated with childhood asthma. *European Respiratory Journal*.

[99] Wang C, Salam MT, Islam T, Wenten M, Gauderman J, Gilliland FD (2008). Effects of in utero and childhood tobacco smoke exposure and *β*2-adrenergic receptor genotype on childhood asthma and wheezing. *Pediatrics*.

[100] Salam MT, Gauderman WJ, McConnell R, Lin PC, Gilliland FD (2007). Transforming growth factor-*β*1 C-509T polymorphism, oxidant stress, and early-onset childhood asthma. *American Journal of Respiratory and Critical Care Medicine*.

[101] Ober C (2005). HLA-G: an asthma gene on chromosome 6p. *Immunology and Allergy Clinics of North America*.

[102] Nicolae D, Cox NJ, Lester LA (2005). Fine mapping and positional candidate studies identify HLA-G as an asthma susceptibility gene on chromosome 6p21. *The American Journal of Human Genetics*.

[103] Choudhry S, Avila PC, Nazario S (2005). CD14 tobacco gene-environment interaction modifies asthma severity and immunoglobulin E levels in Latinos with asthma. *American Journal of Respiratory and Critical Care Medicine*.

[104] Leynaert B, Guilloud-Bataille M, Soussan D (2006). Association between farm exposure and atopy, according to the CD14 C-159T polymorphism. *The Journal of Allergy and Clinical Immunology*.

[105] LeVan TD, Von Essen S, Romberger DJ (2005). Polymorphisms in the CD14 gene associated with pulmonary function in farmers. *American Journal of Respiratory and Critical Care Medicine*.

[106] Williams LK, McPhee RA, Ownby DR (2006). Gene-environment interactions with CD14 C-260T and their relationship to total serum IgE levels in adults. *The Journal of Allergy and Clinical Immunology*.

[107] Eder W, Klimecki W, Yu L (2004). Toll-like receptor 2 as a major gene for asthma in children of European farmers. *The Journal of Allergy and Clinical Immunology*.

[108] Greer FR, Sicherer SH, Burks AW (2008). Effects of early nutritional interventions on the development of atopic disease in infants and children: the role of maternal dietary restriction, breastfeeding, timing of introduction of complementary foods, and hydrolyzed formulas. *Pediatrics*.

[109] Agostoni C, Decsi T, Fewtrell M (2008). Complementary feeding: a commentary by the ESPGHAN Committee on Nutrition. *Journal of Pediatric Gastroenterology and Nutrition*.

